# Adverse or acceptable: negotiating access to a post-apartheid health care contract

**DOI:** 10.1186/1744-8603-10-35

**Published:** 2014-05-15

**Authors:** Bronwyn Harris, John Eyles, Loveday Penn-Kekana, Liz Thomas, Jane Goudge

**Affiliations:** 1 Centre for Health Policy, School of Public Health, Faculty of Health Sciences, University of the Witwatersrand, Johannesburg, Gauteng, South Africa; 2 Health Policy Research Group, Medical Research Council of South Africa, Johannesburg, Gauteng, South Africa; 3 School of Geography and Earth Sciences, McMaster University, Hamilton, Ontario, Canada; 4 Department of Infectious Disease Epidemiology, London School of Hygiene and Tropical Medicine, London, UK

**Keywords:** Social contract theory, Health care access, Suffering, Defaulting, Post-apartheid South Africa

## Abstract

**Background:**

As in many fragile and post-conflict countries, South Africa’s social contract has formally changed from authoritarianism to democracy, yet access to services, including health care, remains inequitable and contested. We examine access barriers to quality health services and draw on social contract theory to explore ways in which a post-apartheid health care contract is narrated, practiced and negotiated by patients and providers. We consider implications for conceptualizing and promoting more inclusive, equitable health services in a post-conflict setting.

**Methods:**

Using in-depth interviews with 45 patients and 67 providers, and field observations from twelve health facilities in one rural and two urban sub-districts, we explore access narratives of those seeking and delivering – negotiating - maternal health, tuberculosis and antiretroviral services in South Africa.

**Results:**

Although South Africa’s right to access to health care is constitutionally guaranteed, in practice, a post-apartheid health care contract is not automatically or unconditionally inclusive. Access barriers, including poverty, an under-resourced, hierarchical health system, the nature of illness and treatment, and negative attitudes and actions, create conditions for insecure or adverse incorporation into this contract, or even exclusion (sometimes temporary) from health care services. Such barriers are exacerbated by differences in the expectations that patients and providers have of each other and the contract, leading to differing, potentially conflicting, identities of inclusion and exclusion: defaulting versus suffering patients, uncaring versus overstretched providers. Conversely, caring, respectful communication, individual acts of kindness, and institutional flexibility and leadership may mitigate key access barriers and limit threats to the contract, fostering more positive forms of inclusion and facilitating easier access to health care.

**Conclusions:**

Building health in fragile and post-conflict societies requires the negotiation of a new social contract. Surfacing and engaging with differences in patient and provider expectations of this contract may contribute to more acceptable, accessible health care services. Additionally, the health system is well positioned to highlight and connect the political economy, institutions and social relationships that create and sustain identities of exclusion and inclusion – (re)politicise suffering - and co-ordinate and lead intersectoral action for overcoming affordability and availability barriers to inclusive and equitable health care services.

## Background

With universal health coverage firmly on the global policy agenda, there is renewed interest in finding ways to alleviate inequities in health care, address access barriers, and make services more inclusive for marginalized groups [1]. Accessible care requires the provision of affordable, available and acceptable services, responsive to patients’ needs and expectations [2,3]. However, health system priorities, values and arrangements are often not well aligned with patient circumstances (especially those from marginalized groups), diminishing their ability to negotiate quality care and fueling inequities in access [2,3]. Closing the social, cultural, financial and/or physical distance between patients and the health system is consequently an important part of making health care more inclusive and equitable [2,3]. Yet this is not easy, even with legal provisions, equity-oriented policies and political will. Additionally, fragile and post-conflict countries also face challenges of rebuilding weak, fragmented or decimated health systems, overcoming historically-entrenched inequities, transforming relationships and changing authoritarian institutions [4]. Twenty years into South Africa’s democracy, the right to access health care is constitutionally guaranteed but access barriers continue to inequitably affect many who experienced the dispossession and structural violence of apartheid - poor, black, rural and informal-urban communities [5,6]- as well as newer marginalized groups, including refugees and migrants [7]. The social contract has formally changed from apartheid to democracy but exclusion, including from health care, continues. A social contract describes institutions, principles and practices that structure and legitimate relationships between citizens and with the state [8]. In this article, we draw on social contract theory to examine how post-apartheid health care is envisaged and practiced by patients (citizens) and providers (state) in the public sector. We consider ways in which identities of inclusion and exclusion are generated through this contract and related implications for conceptualizing and promoting inclusive health care in a post-conflict setting.

### A post-apartheid health care contract

We situate South Africa’s right to access health care as part of a new social contract – initiated in 1994 with the country’s first racially-inclusive elections - to “build a democratic state founded on the values of human dignity, the achievement of equality and the advancement of human rights and freedoms” [9]. This social contract represents a break from the country’s apartheid past of legislated oppression and structured racism. It conveys a Rawlsian “moral vision” in which rights and responsibilities are fairly balanced and justice is served through mutual agreement between citizen and state [10]. In health, this democratic goal is formally communicated through laws, policies and codes of conduct, including the Bill of Rights, Batho Pele Principles (People First), the National Health Act (2003) and the Patients’ Rights’ Charter (PRC). These instruments define rights and responsibilities for providers and patients, and contextualize the health system – with its aspiration to provide equitable, quality care for all - within a wider context of redress [11-13].

However, while a democratic social contract portrays itself as a product of mutual agreement in which rights and responsibilities are fairly balanced, a critical perspective warns that this is not necessarily a “magic formula” [14] equitably serving all members of society [8,10,15-17]. Instead, the democratic ideal may depoliticize and hide structural issues, such as the “political economy, class, income inequity, education, and the bureaucratic compulsion to meet targets” [10], excluding certain people and groups (often those most in need of social assistance) as much as it includes [10,18,19]. In South Africa, two decades after apartheid, an inclusive, equitable society remains elusive. High levels of poverty, inequality, unemployment and “everyday suffering” [20-22] persist [5,22,23]. Barriers to inclusive health care include high transport costs, large distances to services [24], and a public sector that services 85% of the population with just 56% of health expenditure [25]. Rude, dismissive, sometimes abusive, provider actions have also been documented [26,27] and the health system remains fragmented and “dysfunctional” [5] characterized by weak stewardship, a human resource crisis, and limited financial, technical and administrative capacity [5,28]. Furthermore, transformation efforts have been undermined by the country’s crippling, complex disease burden of HIV/AIDS, TB, non-communicable disease (NCD) and violence [5]. Efforts to improve the country’s poor maternal and child health outcomes have also been frustrated by “avoidable” health system and patient-related factors, including a lack of appropriately trained staff, inadequate monitoring of patients, poor teamwork and leadership in facilities, blood shortages, patient delays in seeking care, and limited or poor antenatal attendance [23,29,30]. Looking at the impact of sub-optimal care on maternal deaths between 2008 and 2010, the National Committee on Confidential Enquiries into Maternal Deaths (2012) found that: “In 23.2% of cases the death was thought to have been probably avoidable and in a further 30.2% the death was considered possibly avoidable” [30].

Yet, there are also signs of progress. South Africa’s ART programme - now the largest in the world – is making strides: since 2009, new HIV infections have declined by 63% and the mother-to-child transmission rate has fallen below 5% [31]. There are also efforts to better integrate HIV and TB services, and develop a national NCD plan [23]. Other policy reforms, including reengineering of primary health care and piloting of a National Health Insurance system, similarly signal renewed state commitment to expanding welfarism and implementing an inclusive and equitable health care contract. In this article, we use narratives of those seeking and delivering – negotiating – health care, to explore this contract in practice. In particular, we focus on access to maternal health, tuberculosis (TB) and antiretroviral (ART) services, given that mortality and morbidity in these areas account for a large proportion of the country’s burden of disease [5]. Additionally, HIV, TB and maternal health require sustained engagement with the health system. As they are often closely associated, simultaneous access to more than one service may be required. This may present additional access challenges for patients and providers.

## Methods

### Big and small stories

Narrative research involves listening attentively to voices within stories to interpret the meanings that emerge around events, people and actions; as well as the identities that are produced and reproduced through story-telling [32]. Often intensely personal, narratives are seen as a medium through which social realities speak, hide, contest, order and disrupt, whether as “big” or “small” stories. Big stories, commonly drawn from in-depth interviews and clinical encounters, “entail a significant measure of *reflection* on either an event or experience, a significant portion of a life, or the whole of it…*making meaning*” [33]. They are usually past-oriented and linked to important life events and rites of passage [32,34], such as the onset of illness, initiation of chronic treatment, or the birth of a baby. By contrast, small stories are more immediate and shared, found in everyday talk and local “sites of engagement” [34,35]. Expressed in snippets of interaction, rather than as coherent grand narratives, they are conducive to analyses of contemporary social media, corridor chat and conversations in social settings [33,34]. Without reflexive distance, they are often about very recent, or even upcoming, events, becoming “rehearsals for later action more than reconstructions of the past” [34].

Although big and small stories suggest different levels and entry-points to narrative research, they are not dichotomous and recently, there have been calls to consider the synergies between them [34]. In this article, we draw on both, using in-depth interviews with 45 patients and 67 providers, and field observations from twelve health facilities in one rural and two urban health sub-districts in South Africa. This study was carried out as part of the *Researching Equity in Access to Health Care* (REACH) project, a five-year, multi-method study of equity in access to TB treatment, ART and maternal deliveries in four South African provinces [24,36,37].

### Ethical approval and consent

Ethical clearance was granted by the South African Universities of Cape Town (460/2006) and Witwatersrand (R14/49), and permissions were also obtained from relevant provincial and local health research committees and district- and facility-managers. Informed, written consent was obtained from all individuals interviewed. At a staff meeting, health care workers were invited to participate in facility observations, and signed consent was obtained from managers in observed areas. Written consent was obtained from patients before any consultations were observed. Posters in local languages informed patients and visitors that the facility was being observed for research purposes; a message often reinforced by staff announcing us in waiting areas.

### Telling and documenting stories

Interviews and observations took place between June 2009 and July 2010. We were particularly interested in the challenges faced by both men and women living in rural and urban contexts, who were successfully or unsuccessfully accessing TB, ART and maternal delivery services. Successful service users were patients who were adherent to treatment or who had delivered in an appropriate facility, while unsuccessful users included patients whose treatment had been interrupted or who had delivered at home or before arrival (BBA) at a facility. Therefore, participants were purposively sampled according to these criteria and recruitment followed multiple means, including at facilities (for example, using admissions registers to identify BBAs, approaching patients queuing to see doctors, pharmacists, social workers); via community health workers (often tasked with tracing non-adherent ART and TB patients); and from local HIV/AIDS support groups (attended by a variety of patients – adherent and non-adherent, new and experienced). Providers were similarly selected purposively to reflect different age groups, levels of seniority, and length of service. Patients were interviewed in their home languages by trained fieldworkers, at/outside health facilities, in their homes, or other “sites of engagement” (for example, a coffee shop), where they felt comfortable to talk. We used an interview guide to encourage the telling of illness/pregnancy trajectories and treatment/delivery experiences within the context of everyday life: relationships, support networks, employment, education, and political transition. Providers were interviewed in health facilities about their career paths and understandings of access challenges, as well as their own health-seeking behaviours. Additionally, one focus group took place with twelve community health workers in the rural district, covering similar issues.

Interviews were audio-recorded and lasted between 45 minutes to 1.5 hours of recorded time although there were often delays and interruptions - small stories of daily life – that prolonged each engagement providing important insights that were discussed in weekly team debriefings. Interviews were translated into English, transcribed and reviewed by the research team for questions of clarification and further exploration, and we conducted follow-up interviews with patients we were able to trace (n = 21). All interviews were anonymized and stored on a secure server available only to the research team.

Team members also observed the services in each sub-district, across a few weeks, on different days and at different times. Formally, these observations were non-participatory, recorded as field notes and structured through an observation grid that drew attention to physical space, patient flow, interactions among and between patients and providers, daily tasks, and responses to unusual or problematic situations [38]. Less formally, we were often drawn in to the daily routines - locating and filing records, finding grant-related information for patients, preparing and drinking tea with staff, chatting to patients in queues. Not always written down, we shared these experiences in various, often fragmented, ways - on the drive home, waiting for team debriefings, in response to a newspaper headline or someone else’s experience- thereby generating our own set of small stories nested in the bigger narrative of access to health care.

### From stories to narratives

Data were analyzed by members of the team using Atlas.TI. The analytical task of narrative research is to convert stories into narratives through actively listening to, and interpreting, textual voices. While interpretation starts within each story, it is not devoid of context but rather strives to “make sense of personal experience in relation to cultural discourses […] a window to the contradictory and shifting nature of hegemonic discourses, which we tend to take for granted as stable, monolithic forces” [32]. Stories are thus interpreted *in situ* and are often compared to similarly-located stories to gain a richer understanding of the situation, as well as the identities generated in specific contexts [32]. Our interviews and observations were situated in and around local clinics and hospitals – literal manifestations of the right to access health care and the democratic social contract. Our resultant narratives of inclusion and exclusion from the contract represent but one interpretation. However, while allowing for alternatives, narrative research is not a relativist exercise devoid of methodological rigour or “trustworthiness” [32]. Interpretation needs to be grounded in the texts analysed and tested against other explanations and a-typical cases, to show its feasibility. In our analysis, we embarked on an iterative process of interpretation by collectively sharing and debating our understanding of the stories with reference to social contract theory, and refining them into the narratives presented below. In selecting these narratives, we have tried to give voice to as many participants as possible. However, we recognise that these voices are limited in number and that other notions of inclusion/exclusion might be present - amongst those accessing services, as well as those who are partially- or fully-excluded from the health system as a whole. In recognition of the constraints of second-language translation and for clarity reasons, we have made minor stylistic and grammatical changes.

## Results

Social contracts are clearly symbolic rather than conventional, legal contracts involving reciprocity, mutual consent, terms and conditions [10]. However, contractual rhetoric provides a way of organizing and revealing the moral vision, inclusions and exclusions of social contracts and our results are therefore loosely arranged to explore the terms, expectations, threats and assertions of the post-apartheid health care contract.

### Entering and maintaining the post-apartheid health care contract

In this section, we explore the expectations that patients and providers have of themselves and each other, and their reasons for seeking or delivering health care services: why they enter and remain in the post-apartheid health care contract and what they expect from it.

### Patients

#### In need of care

To become a patient and thereby enter the post-apartheid health care contract, individuals must need care. To establish need, the health system produces indicators, measures and clinical benchmarks that guide diagnosis and treatment. These include guidelines for interpreting physical symptoms, testing blood, urine and sputum, weighing patients, and analyzing their medical histories. In our study, most patients indeed sought care when they felt they were sick, pregnant or ‘in labour’ - in need - presenting with physical symptoms that providers then measured and assessed to determine a treatment course:

I just started feeling my legs were painful, swollen and they were very sore, [I was coughing badly] so I decided to come to the clinic so that they can check it out […] and they ran some tests, they tested me for HIV and found that I was infected and […] have TB. So I had to go to classes where they taught me about the treatment of HIV, after that they referred me to [the ART clinic] they checked me, actually they took my blood, and then they gave me the HIV tablets […] TB tablets as well (female patient, 23, TB/ART services, rural site).

However, needing care from the patient perspective was not always enough to secure patient admission to the health care contract. Some patients *felt* very much in need of medical attention but did not always receive it, even if they expressed their need to providers. This could result in their exclusion, sometimes just temporarily, from the contract, with providers overlooking patient histories, patients confusing (often unexplained) treatment side-effects with ill health, lack of equipment, lost or unfiled records, or false test results.

The clerks would tell people that their results were lost and advise them to have their tests re-done, instead of ever managing to get on top of the unfiled results’ backlog (observation notes, ART services, urban site 2).

My period was gone, I was two months when I found out that I was pregnant […] I went to the [Midwife Obstetric Unit] and they told me that they don’t do pregnancy tests there and they told me to go to a private doctor, and the private doctor did the test (female patient, 29, maternal services (homebirth), urban site 1).

With his second case of TB, a casual construction worker (unable to work at the time of the interview due to his ill health) was told that his sputum tests were negative and to retest in eight weeks:

I said to the sister, “but I can feel that I am [too] sick to wait for eight weeks” so sister said, “you want to have TB?” but I said, “sister, it is not that I want TB but I can feel my heart is sore and I am short of breath” (male patient, 35, TB services, urban site 1).

Conversely, some patients did not realise or acknowledge that they needed (specialized) care until the system ‘told’ them (particularly women diagnosed with HIV during pregnancy and patients who tested HIV positive through the TB service). Sometimes, individuals only entered the health system when they were extremely sick or during/after the birth of a baby; more than simply in need but in crisis.

You still get patients who are very sick, being initiated very, very late in their stages of the illness (female social worker, aged between 30–34, ART services, urban site 2).

The pains started around two […] Then at around three that’s when I woke my baby’s father and when he tried to find transport from our neighbours, we found that their cars do not have petrol. Then we called an ambulance and they were not cooperative […]. Then at around four he called them again and told them that I am ready to give birth now. Fortunately this time the [emergency services] lady answered the phone and she listened. He then explained to her that the baby is coming out now. Then the lady asked if there was an older woman in the house but he told her that there is just the two of us there. Then on the other hand they heard the baby crying. Then the lady asked if everything has come out and he said no […] (female patient, 27, maternal services (homebirth), urban site 2).

Complex, varied reasons were offered for such delays, including stigma and fear of being diagnosed with HIV or TB, high costs of seeking care (see also [24]) and, for six of the eight babies born before arrival, unavailable or delayed ambulances (see also [27]).

In keeping with the narrative of (extreme) need, once admitted as patients to the health care contract, many chronic care participants were motivated to stay by the alleviation of physical symptoms and improved health:

The hospital visits are good for me because I get my treatment and that will help me stay alive for a long time (female patient, 23, TB/ART services, rural site).

[…] reason that made me to continue with the treatment is that I feel comfortable with the treatment and you cannot see it that I am ill. I am no longer coughing or suffering from headaches. I am always feeling well and I have no complaints with my health (female patient, 32, ART services, rural site).

### Complying with the terms of the health care contract: the docile patient

However, from the perspective of the health system, needing care and feeling better with treatment, while important, are not sufficient, for ongoing patient inclusion. Ideally, patients must also take on a medical discourse and internalize their responsibilities:

[Before being initiated onto ART, patients] have to attend three classes. The day they have to initiate, they come to us and there is a form they have to sign that states they do understand that they have to take the medication for the rest of their life. Because sometimes they might come back and say that they didn’t want to take the treatment so by signing the form they are actually binding themselves and showing responsibility (female enrolled nurse, aged between 45–54, ART services, urban site 2).

Pregnant women must similarly show responsibility by following an ideal care pathway: early and regular attendance of antenatal care, pre-booked deliveries, postnatal care, and re-entry into family planning services. In this narrative, to be successfully admitted to the health care contract, patients themselves must become authorities, “experts” [39], about treatment adherence, weight, blood pressure, good nutrition, medication, CD4 counts, viral loads, and other medical touchstones important for their physical recovery or safe pregnancy: “they have to know them by heart” (female enrolled nurse, aged between 45–54, ART services, urban site 2). They have to become self-disciplined [40].

Additionally, patients have to show that they can be trusted to maintain this contract, usually by expressing willingness, commitment and adherence to chronic treatment or natal care (measured by pill counts, improved physical symptoms, blood results). Being compliant, deferring to the provider’s opinion and accepting their diagnosis, treatment path and rules, are also important for ongoing patient inclusion. For many patients in our study, this meant being quiet and passive – becoming “docile bodies” [40] – rather than risking humiliation, ridicule or ostracism:

It’s like the nurse that was helping us [with our antenatal care] had an attitude; when we asked her something, she treated us like children or comics. She was so impatient […] shouting all the time, when we asked for help she would get irritated and you could see that she is irritated. Especially there was another lady here […] who did not understand English, meaning these medical terms they use […] they made her a joke […] All the nurses were laughing at her […] You do understand that this lady didn’t know anything, that is why she was asking because she doesn’t know anything like that […] once when I was trying to ask something as well and this nurse just looked at me as if I was crazy and never responded, so I just kept quiet (female patient, 25, maternal services (stillbirth), urban site 2).

Here, belittlement and public shaming of “ignorant” or “troublesome” patients by providers – a commonly reported practice in this study and previously [26,41,42]– serves as a form of contractual maintenance, keeping docile those who witness the public spectacle, while simultaneously reprimanding transgressors [40]. In Foucauldian fashion, successfully included patients were often also rewarded with greater autonomy and independence *viz.* fewer clinic visits, self-management of larger volumes of treatment drugs, and other incentives to maintain their contractual commitment:

you have to come to the clinic every time to take the pills but if you [show that you] know how to take the pills […] they give you more pills to take on your own […] when they see that you are committed into taking your treatment (male patient, 36, TB/ART services, urban site 2).

### Providers

#### Moral guardians of the post-apartheid health care contract

As an expression of the right to access health care for all, the post-apartheid health care contract has to be inclusive of everyone; no one in need of care should be excluded by having their treatment or care denied. This rhetoric was often repeated by providers in our study:

Remember that you don’t require an ID or passport. Everybody gets treatment, no matter their creed, race, nationality (female programme manager, 50, ART services, urban site 2).

So we know we have been told to treat [patients] equally irrespective of their social standing or whatever (female assistant manager, 61, maternal services, urban site 2).

However, with the onus falling on patients to prove their worthiness for inclusion, this all-inclusive ideal was rendered conditional in practice. Not everyone in our study was automatically or un-problematically included. Simultaneously, the creation of conditions for patient inclusion in the health care contract conferred a form of moral guardianship on providers: contractual caretakers who ultimately determine which patients to include in what ways. For many providers in our study, this guardian role was expressed as one of paternalism, reminiscent of an era before patients had the rights they do now [41]:

You know, patients are like children. A child does not know a thing. You as an adult, you teach the child. The patient knows nothing about TB. He knows nothing about what he is suffering from. We’ve got all the knowledge and the facts about TB (female operational manager, aged between 50–54, TB services, urban site 2).

Often, steering patients on the “right” path, teaching them to do the “right” thing, was tied to an identity of working altruistically, “in service” of patients.

#### Altruistic professional versus uncaring mercenary

With their job descriptions and place in the system, health care workers are - by definition – included in the health care contract. In our study, many spoke about becoming providers and thereby entering the contract in response to a “calling” or willingness “to serve”; a vocation. For some, this was an active political decision to uplift their communities and support the country’s transformation agenda. However, others admitted more pragmatic reasons for initially joining their profession, mostly linked to limited career opportunities, either when apartheid policies restricted career paths (especially for black women) [43], or post-1994, where training is state-subsidized. Yet, most followed with: *but*, “I have learnt to love nursing and think it is a calling in a way” (female programme manager, 50, ART services, urban site 2), deferring to the dominance of an in-service narrative:

Currently, I am in public service because it’s part of the training and I am specialising in obstetrics, so […] I am forced to be part of the service. [But] I intend to stay longer even after qualifying as a specialist because it’s eh, I think the public sector has more people who need us than private: people who are not cared for, people who have less resources who cannot afford the private doctor, so they have got nowhere to go. And they need us most. So I believe it will be the right thing to be where l am needed actually […] where people, the poorest of the poor, are needing a doctor (male registrar: obstetrics, aged between 35–44, maternal services, urban site 2).

This envisaged ideal was given further definition by providers who spoke about expected ways of being and interacting once in the provider role - caring medical experts and *more*: professionals, conducting themselves “properly”, maintaining confidentiality and establishing boundaries with their patients:

[My] problems are left behind, put them aside because […] the patients […] too have their own challenges in life and part of those challenges are the reason why they are here and they want us to help them, so it won’t be professional for me to offload to them as a service provider (female VCT counselor, 28, TB/ART services, urban site 2).

“Being professional” was also linked for providers to punctuality, neatness, institutional loyalty, supporting and respecting colleagues, and showing willingness to go the extra mile; a reminder that providers operate and are accountable within a complex organisation, governed by formal employment contracts and codes of conduct. This requires that providers respond to a set of expectations from within the health system (alongside acting in the service of patients and communities) in order to maintain their own inclusion in the health care contract. They too must become “docile bodies” [40].

Some patients in our study similarly imagined an ideal provider motivated by altruism and professionalism. But, this expectation was often revealed only in contrast to a disappointing or negative experience:

[…] the staff here is useless because *they don’t do their work like they love what they do* (patient comment, observation notes, ART services, urban site 2, emphasis added).

And even the way they were treating us it was *not a proper way* of treating other people. We don’t deny that we are there to get help but that is *not the way they should* offer us their help […] they don’t respect us at all, it’s like we are there to bother them, some of them it seems like they bring their problems from home to work, you can’t wait to be out of the clinic sometimes because of the way they treat people (female patient, 25, maternal services (stillbirth), urban site 2, emphasis added).

Other patients had lower expectations of health care workers, repeating a popular public stereotype of providers as uncaring and mercenary, acting in their own interests, rather than in support of patients or communities [44]. Many providers, especially nurses who had been working in the system for a long time, were also aware of, and reacted to, this stereotype:

[When community members see you are a nurse] they suddenly forget that you are one of them […] I usually take off my epaulets the minute I reach home. I go back into my ordinary clothes and be just like the basic person that is accessible to them (female assistant manager, aged between 55–64, maternal services, urban site 2).

[…] after work, I’ve got a spare jacket in my car. I take off this [gestures to jacket with epaulets] and wear my spare jacket because people no longer respect nurses like before. They say nurses are not nice, they are very bad. I don’t know why (female professional nurse, 60, maternal services, urban site 2).

Such representations of providers (particularly nurses) as “not nice” or “very bad” contradict and threaten their stated raison d’etre of altruism and professionalism, and many nurses in our study expressed hurt, anger, demotivation, and even withdrawal from the contract as a consequence. While most providers identified positive changes with democracy, some (mostly older nurses) also mourned a lost identity; on one hand nostalgically evoking a sense of belonging to an earlier social contract which had been configured - in a highly complex way around race, class and gender - to accord nurses a certain social status within their communities [43], and on the other, conveying a sense of displacement, of not quite belonging to the present social contract and feeling marginal in the changed context.

#### Threatening the post-apartheid health care contract

Veitch (2011) argues that those within the margins of society are not marginal in the discourse of the social contract but rather generate anxiety and attention because they have *potential* to affirm (if they can be re-included) or derail (if they refuse to participate) the moral vision of an inclusive and equitable contract, and the structural, systemic divisions that this ideal may gloss over [10]. In Foucauldian terms, the social contract produces “deviant categories” of people – hazardous, useless or worthless – who simultaneously threaten and entrench the status quo and the docile bodies it safely includes [10]. In this section, we consider patient and provider accounts of access barriers to health care and ways in which these threaten the post-apartheid health care contract.

#### Defaulting or delayed: the dangerous patient

Drug resistance caused by patients interrupting their ART or TB treatment (defaulting) has undermined the TB cure rate and compromised the efficacy of ART, creating enormous frustrations for providers, patients and the South African health system as a whole [45]. Similarly, the delayed presentation of pregnant women to antenatal care is a major challenge for improving the country’s poor maternal health outcomes [29]. For providers in our study, defaulting or delayed patients were generally represented as dangerous because of their risk to patient- and community-health but also, because they were perceived to threaten provider professionalism: “when they have defaulted it comes back on you” (female student nurse, aged between 25–30, TB services, urban site 2). Defaulters draw unwanted attention to clinical services (and a related “bureaucratic compulsion to meet targets” [10]) and thereby challenge the quiet inclusion of providers in the healthcare contract:

Now remember that if your TB cure rate goes down, [the authorities] will phone the manager of the clinic to say, “hey there is something wrong with your services?” Now if you don’t go in and pick up the areas of concern and address them you are not going to be able to account for your low cure rate, you know (female operational manager, 54, TB services, urban site 2).

I get affected when I have defaulters because they put a drawback on me and I can’t push [up] my cure rate […] I look like I am not taking my job seriously, you see, something like that. And it looks like I don’t give health education but you know that you do give them health education (female auxiliary nurse, 53, TB services, urban site 2).

Staff in maternal services similarly expressed anxiety and frustration about having to manage complications (and associated high mortality and morbidity rates) that might have been avoided if women entered the health care contract earlier in their pregnancies.

Various explanations were offered for defaulting and delayed attendance of antenatal care services, including HIV-related stigma, lack of social support for patients, and patient beliefs and pressures:

When a patient is sick, this is now culturally speaking, they will first try other things, you see, before they come to hospital. As a result of that, the greater majority of the patients who die usually come late to hospital, very late. And now with HIV around, it's even worse. And sometimes, while patients are still in hospital the relatives will come and say “look, we would like to have our patient because we have established what the problems are, we need to take the patient home” (male doctor, age unknown, TB services, rural site).

Treatment side effects, health improvements with treatment or simply wanting a break from the chronic routine were also presented as reasons for defaulting:

When they start to feel better they will give you trouble (female auxiliary nurse, 53, TB services, urban site 2).

This lady was on treatment since 2004, then she just said, “I just wanted to take a break and um I mean I’ve been taking this medication far too long now and I felt that I was better […] so I just wanted to take a break” and actually when she came back [six to eight months later], she was very sick (female social worker, aged between 30–34, ART services, urban site 2).

Some defaulters were portrayed as “stupid” or “ignorant”:

Patients are so ignorant because honestly and truly they are educated on this and all the information is all around them but because of blind ignorance […] they end up saying, “I’d rather die than go to the clinic to take treatment” (female student nurse, aged between 25–30, TB services, urban site 2).

Others were presented as defaulting out of entitlement, wilfully abusing their contractual rights without living up to their responsibilities “because if one feels that they want to stop treatment they do so”:

Before people had rights, patients used to do the right things and you wouldn’t fight or argue with them, everything was nice (female auxiliary nurse, 53, TB services, urban site 2).

I hate the patients’ rights with all my heart. Because they have got rights and they ignore the responsibilities […] And you can’t do a thing, your hands are tied (female operational manager, aged between 50–54, TB services, urban site 2).

### Poverty: the suffering patient

Poverty was identified by both patients and providers as a significant barrier to uninterrupted patient inclusion in the health care contract, impacting especially on transport affordability, particularly difficult for those in the rural area, where distances to health facilities are large – see also [24]) and the availability of food for patients (essential for the efficacy of ART and TB treatment):

[Defaulters] don’t have money to go to [the clinic], nê? At the same time they don’t have food at home, they don’t have money to buy soap to wash (female staff nurse, 28, TB services, urban site 1).

Most patients in our study wanted to be adherent, often making large sacrifices in an effort to remain included in the health care contract - walking long distances daily (often in bad weather while in frail health), waiting for a number of hours in long queues, borrowing medication or money, sometimes even selling assets (see also [24]) - and consequently feeling that if they had to break the contract, this was not done by choice:

I stopped in the middle because these tablets you cannot take them without eating. And I was not working and these tablets are eating your stomach and [you vomit] when you have not eaten. I tried to borrow some money from someone to sell some cheap stuff at home so that I can eat because even my children were not working - they were looking to me [to put food on the table]. That is why *I look like someone who is taking treatment wrongly* [i.e. defaulting]. Then after I sold those things, then I saw the profit; there was food in the house then I always came in here […] So when I started having some money I always came to the clinic always (male patient, 53, TB services, urban site 1, emphasis added).

Many providers were sympathetic to such “everyday suffering” [20-22] and did not allocate blame: “at the end of the day, you know that [patients] can't take medication on an empty stomach” (female pharmacy assistant, 26, ART services, rural site). Rather, this suffering was largely viewed as beyond the control of patients, providers and the health system; a social determinant of health requiring upstream and long-term solutions:

Maybe to get them a job so that they can get something to eat, because most of them, they are not working, there’s no pay. If maybe, the government can do the pay [grants] for them […] (female acting TB assistant, 27, TB services, urban site 1).

In addition, however, many patients in our study *included unacceptable provider actions and attitudes* as exacerbating their suffering.

### Unacceptable care: the dangerous provider

In our study, patient inclusion in the health care contract (mostly from the patient perspective) was threatened by unacceptable provider behaviour and the delivery of uncaring care. In some extreme cases, negative interactions with providers resulted in patients withdrawing (even if temporarily) from the contract by dropping out of services:

[…] the nurse was standing in front of many people talking to me loudly saying that my CD4 count now is very low and I have to start the treatment afresh. And that hurt me a lot because *she should have been* polite, maybe call me to her office because she’s got one, and talk to me nicely, explain that because I have defaulted and my CD4 count is low, then I have to start the treatment afresh, *unlike what she did* shouting at me in front of many people. Then I went home but I was not ok with that. Then after two or three days I came back here and told her how I feel about what she did. And she only shouted back asking me why am I there at that time to collect the pills and that’s when I decided to give up the pills [for eight months until I developed sores. Then I forgave the nurse and went back to the clinic] (male patient, 35, TB/ART services, urban site 2, emphasis added).

More typically, chronic patients continued (or attempted) to access services but were left feeling disrespected, dehumanised and devalued in these interactions. Some maternal patients similarly narrated negative experiences with health care workers during antenatal care and labour:

I remember this one nurse as I was screaming in pain she just walked out and the other one was sitting on the corner with her phone and her feet on top of a table, laughing and chatting […] I started banging the wall because I couldn’t bear the pain. She just said to me I shouldn’t do that because I will break down the hospital. The other one said to me “you are here to bother us” and I responded that the only thing I’m asking for is water - they still said I’m not supposed to drink water (female patient, 26, maternal services (stillbirth), urban site 2).

While never questioning their own commitment or intention to serve patients, certain providers in our study did reflect on the behaviour of a few “difficult” colleagues:

[She is] somebody who is difficult […] She does not see the perspective [of the mothers or the hospital’s efforts to entrench a new baby friendly, kangaroo care policy]. We are struggling [to manage her] (female assistant manager, 61, maternal services, urban site 2).

Dismissive, overly-technical or limited communication with patients was also identified by providers as a challenge for sustaining patient inclusion:

[Ongoing access] depends on the way we as providers treat them [patients] and how well you communicate with them (female VCT counselor, age unknown, TB services, urban site 2).

### Under-resourced: the overstretched provider

For providers in our study, unacceptable care was largely narrated around the negative actions and attitudes of *individuals*. In contrast, resource constraints and staff shortages emerged as more systemic threats to the quality of care delivered and, ultimately, their own inclusion in the contract:

I would like to see the clinic being well staffed so that we can give quality nursing. What we are doing now is not quality nursing. We are just trying to cover everybody; nobody must go home having not met the nurse. And we don’t feel good about it. We are not practising what we are trained to do but it’s beyond our powers. […] When I see a crisis there, I turn that way, another crisis there, I turn that way. There is no planning (female operational manager, aged between 50–54, TB services, urban site 2).

[…] the clinic is growing, it’s not frozen [unlike staff posts] and the work must go on, you know because you don’t want the patients to be unhappy so […] we try to make the best of you know the few resources but of course, I mean I woke up today I was sick but [came to work nonetheless because …] we see over 120 patients in the clinic and then [I am the only] doctor, it’s crazy (male doctor, 36, ART services, urban site 1).

Feeling overstretched and under pressure, yet also beholden to “keep patients happy”, was a common complaint for providers, often causing conflict, rather than complementarity, between being an accountable employee responsible for “getting the work done” on one hand, and acting “in service of patients”, on the other: “at the end of the day it's not the best quality of care that you are supposed to [deliver]” (female professional nurse, age unknown, TB services, rural site). Through this narrative, providers perceived themselves as “doing their best” under difficult circumstances (much like “suffering” patients). Any compromised patient care was presented as beyond their control and despite their best intentions as guardians of the contract. Many providers felt highly anxious and frustrated by having to manage competing and intense pressures within the constraints of limited resources, further fueling a sense of marginalisation from the present context:

I lie awake at night, worrying that I will be sued, my house and car repossessed, my family compromised by these challenges. And I think, “why don’t I just emigrate or leave the public sector?” (manager comment, observation notes, maternal services, urban site 2).

Demotivation, burnout, and withdrawal from the system are ever-present threats on the provider-side of the health care contract. Patients in our study were not unaware of these challenges but felt they should not have to bear the negative consequences thereof:

I felt that I was not treated the way I wished I would have been treated. I know it is not a private hospital and I understand [providers] are overworked and underpaid but it is not my problem. It should not affect my health (female patient, 26, maternal services (stillbirth), urban site 2).

### Reasserting the post-apartheid health care contract: ways of including

From a Foucauldian perspective, the social contract seeks to reward and re-integrate those on its margins (defaulting or suffering patients, overstretched or difficult providers) when they comply with contractual norms and return to the mainstream, while denying access to those who stay out of the contractual reach [10,46]. In this section, we consider some ways in which the post-apartheid health care contract strives to reassert itself when threatened by access barriers, including poverty, resource constraints and unacceptable service delivery.

Trusted, docile patients and quietly productive providers – the successfully included – are, in many ways, invisible in the health care contract, while defaulters from chronic care and late-comers to maternal services, along with the providers responsible for them, solicit much more attention. For ART and TB services, the health system has developed extensive procedures and protocols for trying to re-include defaulters: follow-up telephone calls, home-based visits, making use of treatment buddies, volunteers and community health workers, sometimes even nurses themselves, to trace people:

[Our TB nurse] was so dedicated, she would go out to look for a patient that hasn’t attended to for a number of days […] personally she would go out and find the patient ask, tell the patient that she didn’t see her in the clinic […] beyond the call of duty (female student nurse, aged between 25–30, TB services, urban site 2).

### The caring provider

Within the South African health system, untraceable patients are usually categorised as “lost to follow up” and excluded, with little further effort to find them. However, for those on the margins in our study (traced defaulters or those who seem about to default), a number of strategies were deployed to retain or return them to care. At an individual provider level, these included positive rewards such as offering encouragement, friendship, food and financial assistance:

We’ve had to give money […] privately […] to patients, which we don’t encourage but sometimes you hear their stories, you just cannot hold back (male doctor, 36, ART services, urban site 1).

Providers also sometimes mediated health beliefs and cultural practices for patients at risk of compromising the efficacy of their ART or TB treatment or defaulting in favour of traditional medicine:

I also tell them that, “people you can do your traditional things but please ask them not to make you vomit […] They can steam you or do anything but they should not make you vomit or give you a laxative because when they do, all that they are getting rid of is tablets in your blood system, you see” (female auxiliary nurse, 53, TB services, urban site 2).

I just tell them that, “if that is what you believe in then its fine” because I can’t stop them from believing in what they want to […]. I just explain to them that sometimes HIV can become latent and undetectable and the person will start believing that they are cured whereas they are not, that person can still infect the other, without forcing them to believe in what you say, they have to make their own choices (female VCT counselor, age unknown, TB/ART services, urban site 2).

### Respectful communication

The value of “good communication” was acknowledged by providers in both chronic and maternal services; important for retaining patients in the health care contract, reasserting the terms of engagement, and even expanding the scope of such inclusion to additional or future services:

I think communicating with the clients is very, very good. You know talking to them even for a short while means you are part of them: “remember that we are part of the community” […]. It’s a matter of marketing our services that we are rendering at our catchment area so that they know […]. One day you want to talk about cleanliness, one day you want to talk about children, you know, you want to talk about the road to health (immunization) card. You know, just to remind them that these are the things *we expect you to do at the clinic* (female operational manager, 54, TB services, urban site 2, emphasis added).

For patients, respectful, attentive communication, humour and kindness positively enhanced their access experience (in contrast to the alienating and disempowering effects of adverse engagements). Often this alleviated their suffering and became a reason to regularly return to services:

I started to know the sisters that helped me and I started to talk with them. They would ask you, “how do you feel? […] what can I tell them about the illness?” […] They helped me a lot. Sister [name] was my pillar here. She was making me happy, she always smiled and she encouraged me to say that it was worth it to live. It was a pleasure to come to this clinic (male patient, 49, TB services, urban site 1).

### Facility-level leadership, flexibility and initiative

Beyond individual provider actions of kindness, facility-level initiatives, driven or supported by managers, were also in place to overcome access barriers and threats to the health care contract. Some of these were consistent with national policies and approaches, such as the ongoing education and training of patients (always potential defaulters) and the implementation of policies aimed at improving quality of care and acceptable service delivery, for example improving the baby-friendliness of hospitals. Other strategies of patient retention were more directly reliant on the initiative and flexibility of senior staff and managers. For example, at one ART clinic where transport “used to be a problem” for many unemployed patients living in an impoverished area eight kilometres from the service, the facility manager arranged for use of an inter-clinic bus previously reserved for x-ray patients, as a way to cut out transport fees. Additional efforts to tackle obstacles included managers seeking donations of food parcels, growing vegetables for patients on site, hosting or partnering with non-governmental and community-based organisations, facilitating patient applications for disability grants, and using referral systems, social workers and counsellors to strengthen available social infrastructure and support.

Individual and institutional efforts were similarly described to manage and contain hostile or uncaring providers and an under-resourced working environment. These included the provision of regular in-service training aimed at imparting “professional values” to providers; constantly reallocating resources, such as linen, medical supplies and staff, between zones of crisis in a facility “we rob Peter to pay Paul” (female assistant manager, 61, maternal services, urban site 2); and encouraging formal and informal support between colleagues and across facilities:

This is a very stressful department. We don’t have formal debriefing [anymore because we ran out of funds]. If a staff member gets to a point with a patient where they just can’t take it, they go to the kitchen so that they can rest […] none of the staff should feel imprisoned. [We run our own debriefing sessions]. The doctor is a priest, so if the staff need to, they can talk to him on site (female programme manager, 50, ART services, urban site 2).

Providers also partook in small rituals, such as a daily morning prayer - typical at most facilities in the country – which seemed to encourage their sense of belonging and reinforce the vocational nature of their work (see also [47]). Provider inclusion was similarly nurtured through informal but institutionalised involvement of staff in personal networks and lives of colleagues: gossip, baby showers, funerals, weddings, birthdays:

Observer 1: [The facility manager] calls for a meeting [to discuss three things outstanding from yesterday’s agenda]: birthdays, farewells and baby showers. They start with deciding what would be the right amount [for staff to contribute financially] R50 or R30 (US $5-3)? A heated debate ensues with some arguing that R50 is a lot of money […] put the matter to a vote again and then after a long time decide that R50 is the right amount. A question is asked about what to do on birthdays […] one staff member presents an example to say that what they did for Valentine’s Day was everyone contributed R5 for cakes.

Observer 2: This meeting is the most animated and involved I’ve seen the staff all day! (observation notes, ART services, urban site 2).

## Discussion

Although access to health care is a right guaranteed for all in South Africa, access barriers continue to undermine this right. In our study, the constitutional ideal of automatic and positive access for everyone, i.e. unconditional inclusion in a post-apartheid health care contract, was contested by everyday patient and provider stories of seeking and delivering health care. Through these stories, we identified a number of barriers to TB treatment, ART and maternal services, including poverty, the nature of illness and treatment, difficult or uncaring providers, an under-resourced health system, negative provider stereotypes, and a “compulsion to meet bureaucratic targets” [10], with related pressures for providers to be accountable employees. In narrating these barriers, participants revealed differing insights and expectations of the health care contract, and with this, differing, potentially conflicting, identities of inclusion and exclusion: defaulting versus suffering patients; uncaring versus overstretched providers.

To be successfully included in the post-apartheid health care contract, patients had to earn and maintain their position by demonstrating that they were sick, responsible and docile, adopting a specific form of patient identity that was often rewarded with autonomy and independence, as well as improved health. Without this identity (which for many patients, was closely connected to whether they had financial means to regularly attend services or afford food with which to take their treatment), patients risked being humiliated or even excluded from services. With a narrative of defaulting, providers described excluded and dangerous patients, dangerous to the population and their own quiet inclusion in the health care contract. Providers also described their efforts – as guardians of the contract - to re-include defaulters. These efforts were linked to institutional pressures to improve cure or retention rates and be accountable employees, as well as to serve patients. Yet, in the resource-constrained environment of the health system, many felt overstretched, unappreciated and unable to consistently deliver good patient care (see also [48]) not excluded but also not safely or positively included – “adversely incorporated” [49] - in the health care contract, and for some, marginalised from the broader social contract through a perceived fall in the social status of nurses.

With a counter-narrative of patient suffering, the financial struggles, competing responsibilities and daily hazards of living emerged as barriers to access [21,50]; social determinants that generate similarly insecure forms of inclusion or even (temporary) exclusion from the health care contract. Although many providers were sympathetic to patient barriers of poverty and difficult life circumstances, for patients, suffering was more than adverse socio-economic conditions. It was exacerbated by uncaring, hostile or dismissive providers and certain health system arrangements, which were often intended to curb defaulting, yet added to the risk of patients dropping out of the system. Conversely, caring, respectful providers and facility-level efforts to alleviate affordability and availability barriers assisted positive patient inclusion in services.

## Conclusions

Establishing individual, population and social health in fragile and post-conflict societies requires not only overcoming past and present barriers to care, but negotiating and implementing a new social contract. Conceptualising access to health care as a social contract, a negotiation between two parties, helps to reveal the relational, constructed nature of patient and provider identities: *created*, as well as reflected, in the social exchanges of health care delivery [51]. Closing the distance between patients and providers, while not an easy policy task [52], may potentially transform these relationships and shift identities of exclusion and insecure inclusion to more positive forms. Because the expectations, threats and terms of the health care contract differ depending on ‘where’ an individual is positioned and ‘who’ they are, jointly engaging citizens and state representatives (including health care providers, managers and policy makers) to re-examine this contract may contribute to narrowing gaps in access. Such engagements could potentially be facilitated through existing legislated advisory and consultative bodies, *viz.* Clinic Committees and Hospital Boards, which formally bring together community representatives, civil society structures and service providers [13,53,54]. While these bodies presently face a number of challenges, including inadequate resourcing and lack of capacity [53,54], the current emphasis on NHI policy reform has seen renewed interest (including from donors) in community participation and governance [53,55] and thus presents an opportunity for reinvigorating such sites of engagement and piloting of patient-provider dialogues at a local level. Additionally, renewed policy interest in management strengthening and health worker training offers an opportunity to advocate for an expanded curriculum that engages with these different perspectives and access challenges [55]. Materials could be designed to explore – through a facilitated process - the differing expectations and identities attached to the health care contract: debating and problematising stereotypes of “defaulting” patients and “uncaring” providers, promoting examples of “caring” providers and “responsible” patients, exploring ways for providers to account to patients, communities and each other, encouraging patient agency, and better-rewarding providers who deliver patient-centred care.

Alongside surfacing challenges of (un)acceptable care, social contract theory also allows for a re-examination of cost and physical barriers to accessing and delivering health care services. Many of these barriers – poverty, suffering, resource-constraints – extend beyond the scope of the health system, excluding people not only from health care but from a wider democratic social contract. Yet, the health system is well-positioned to co-ordinate and lead intersectoral action for social reconstruction (nationally and locally) and should be better integrated with transitional justice processes in fragile and post-conflict states [56]. Additionally, critical social contract theory warns against a retreat or weakening of civil society with a contractual shift from authoritarianism to democracy. Holding the state accountable and making health matter requires coordinated, robust social activism [13], alongside academic inquiry and evidence-based research. Furthermore, rendering visible and connecting the political economy, institutions and social relationships that create and sustain identities of exclusion and inclusion - a (re)politicization of suffering – may offer new policy insights and opportunities for better “rehearsing” [34] a future that is more equitable and inclusive than the stories in our study currently anticipate.

## Competing interests

The authors declare that they have no competing interests.

## Authors’ contributions

BH conceptualised and wrote the first draft of the manuscript, which was critically reviewed by JE, JG, LPK and LT. All authors contributed to subsequent drafting and editing of the manuscript. BH, JE and LPK contributed to the study design and data collection. All authors read and approved the final manuscript.
